# The career identity of young working fathers in dual-earner relationships: A family-relatedness of work decisions perspective

**DOI:** 10.3389/fpsyg.2022.908974

**Published:** 2022-09-15

**Authors:** Anne Crafford, Eileen Koekemoer

**Affiliations:** Department of Human Resource Management, University of Pretoria, Pretoria, South Africa

**Keywords:** family identity, career identity, working fathers, dual-earner couples, role salience

## Abstract

**Introduction:**

Taking on an identity lens, we explore how young working fathers (in the establishment phase of their careers), experience their careers in the context of their changing family roles (shifting ideologies of fathering). We propose that working fathers’ work experiences, work decisions, and career identity are the product of social and cognitive processes in a dual-earner relationship.

**Materials and methods:**

This qualitative study was conducted using an interpretive, and qualitative survey. The data was collected amongst a purposive sample of 45 young South African, well-educated, working fathers, using semi-structured interviews, until data saturation was reached. Interviews were recorded, transcribed, and analyzed using thematic analysis.

**Results:**

The three main themes extracted from the data were: “the meaning of family identity,” “the impact of family identity on career identity,” and finally, “the types of negotiation scenarios” used by working fathers in dual-earner relationships, and how they balance the work-family challenges they face.

**Conclusion:**

This study provides strong empirical support for the family-relatedness of the work decisions perspective, as we highlight the roles of working fathers as indicative of their family identities, and how these then influence their career decisions. Furthermore, our findings shed light on how dual-earner couples negotiate their work-family needs to foster positive work-family outcomes.

## Introduction

Due to the increase in dual-earner couples, the growing number of women in the workforce, and the blurring of boundaries between work and non-work domains, researchers have tried several different approaches to understanding how men and women are combining career with personal life (see overviews in Casper et al., 2018; Bagdadli and Gianecchini, 2019).

In the recent 50-year review of integrating careers and work-family research published in the Journal of Vocational Behavior, Kossek et al. (2021, p.1) explain that “most empirical articles take on a *trade-off* lens approach, assuming an incompatibility between high dual role investments in career and family,” and as such, a gendered siloing of work-family and career issues are portrayed. However, according to Masterson and Hoobler (2014), the context in which work and family decisions are made, is significant. Therefore, emerging researchers, scholars, and practitioners must acknowledge that the way individuals (particularly those in dual-earner relationships/couples) construe their family identities and the meaning they derive from such identities, influences their decision-making both at home and in the workplace.

When considering gender equality in heterosexual relationships, men have conventionally fulfilled the breadwinner role and women, that of homemaker (Lyness and Judiesch, 2008). However, this situation has changed, as more women are entering the workforce (Bird and Schnurman-Crook, 2005), and the gap between women and men in daily participation in housework and childcare responsibilities, is narrowing in the various family types. While much research is focused and available on the changing roles of working mothers and the management of their career and family responsibilities (Araujo et al., 2015; Mkhize and Msomi, 2016), far less empirical studies are focused on how perceptions of working fathers have changed. As fathers are becoming more involved in their fathering role (Lupu et al., 2017), accompanied by their increased childrearing responsibilities, a change in the meaning and value placed on fatherhood is expected (Ladge et al., 2015). This is especially true for fathers with younger children, as this implies increased responsibilities and demands. Literature on working fathers seems to emphasize the time fathers spend on family and work roles (Fong and Bainbridge, 2016), the saliency of the roles they engage in (Erdogan et al., 2019), and their father identity (McLaughlin and Muldoon, 2014). Humberd et al. (2014), more specifically, indicated how men hold multiple images within their father identity, that can reflect various meanings, from traditional to more involved fathering. In this sense, research explains how fathers’ family identities are shaped and influenced by the organizational context they work in and their family situation (Humberd et al., 2014; Ladge et al., 2015). Although this new “involved father” identity is documented in recent literature (McLaughlin and Muldoon, 2014; Pasley et al., 2014; Goldstein-Gidoni, 2020), little is known about the reverse of the situation, i.e., *How does this involved fathering role shape their career identities, work experiences, career progression, or adult career development?* Understanding this question will not only fill the gaps in our knowledge of working fathers’ career and work experiences, but will also shed more light on one social context (dual-earner couple) influencing the adult career development of young working fathers, such as those in the establishment phase of Super (1980). Moreover, this is in line with the call of Greenhaus and Powell (2012) for more research on the influence of family situations on work decisions across life stages. More specifically, the career identity dynamics of young working fathers remains a topic which warrants further research (Ranson, 2001; Ladge et al., 2015).

This study takes on an identity lens to explore how young working fathers (in the establishment phase of their careers), experience their career and work experiences amidst the context of their changing family roles (shifting ideologies of fathering). Drawing upon identity theory, family-relatedness of work decisions (FRWD), career construction and self-construction theory, we propose that working fathers’ work experiences, work decisions and career identity are the product of social and cognitive processes taking place in the context of their family life (Gasper, 1999; Savickas et al., 2009). This implies the need to understand the social context of being in a dual-earner relationship. With this study, we hope to unpack how working fathers’ salient family roles shape their overall sense of meaning in their careers and provide a more nuanced explanation of the identity dynamics, working fathers experience during their career development. Specifically, we seek to contribute to the young adult career development literature in the following ways: (i) expand our understanding of the way in which working fathers specifically construe their family identity and roles, within the context of dual-earner relationships (ii) illustrate how family identity influences the career identities of working fathers and (iii) describe the means through which entangled strands of working fathers’ personal, family and work life are negotiated socially, within the family context (i.e., dual-earner relationship).

### Theoretical foundations

#### Family-related career sensemaking (and family-relatedness of work decisions)

Since the inception of vocational psychology and career counseling, one topic, which has been prominent, is career decision-making (Byington et al., 2019). This stream of research is dominated by educational and occupational choices, and decisions concerning any aspect of the work-life dynamic (Xu, 2021). However, far too little attention has been given to the process of how various work decisions are made, and the extent to which family situations are considered when making them. To kindle research that captures the diverse effects of family situations on decisions in the work domain, Greenhaus and Powell (2012) put forward the notion of FRWD. These authors define FRWD as “*the extent to which an individual’s decision-making process and choice of course of action in the work domain are influenced by a family situation in order to foster a positive outcome for their family*” (Greenhaus and Powell, 2012, p247). This concept is based on the premise that a strong identification with family role relationships will strengthen the impact of the family situation on a work-domain decision, to such a degree that individuals are more likely to consider family situations when making a work-related decision. Thus, individuals are likely to consider family situations when making work-domain decisions to produce a family outcome that is consistent with their values, and how they construe their family identity. According to the FRWD, such a view outlines two important points: (1) the family-related decisions men and women make at work are highly dependent upon the way in which they construe their family identities (Greenhaus et al., 2012), and (2) such decisions are likely not made in isolation, but rather in coordination between partners within dual-earner couples (Challiol and Mignonac, 2005).

Individuals undeniably differ from each other regarding the degree to which they actively carry out explorations, and make their own choices, regarding the values and norms that decide their behavior (Meijers, 1998). As such, family-centric individuals may prioritize families over their careers, while career-centric individuals may focus more on advancing their careers (Frear et al., 2019). Yet, Hall et al. (2012) found that career-orientated individuals can navigate high commitments to careers at the same time as high commitment to family, and still achieve objective and subjective career success. According to these authors, successful employees are those people who manage to find their own ways, over time, to craft careers that work, and that fit what they value most in their family lives. Thus, in line with the principles of FRWD, the argument can be made that (we believe that) individuals’ decision-making processes, actions and outcomes of their careers are influenced by their family situation (or their family identity).

Lysova et al. (2015) agree that individuals who face decision-making situations in the workplace rely on their identities, and the associated rules and procedures for choosing a specific course of action. In this regard, identity serves as a frame of reference for interpreting a situation (Weick, 1995). According to Lysova et al. (2015) the identity in one’s family domain is likely to shape how individuals make sense of their careers and will influence how employees maintain or reinforce their identities. In this regard, individuals seem to turn to their non-work identities and significant others, as a source of meaning to tie these non-work identities to their work (Ramarajan and Reid, 2013).

#### Career identity and career construction

With individuals having to take increasing responsibility for their own employment and career development (Hirschi, 2018), the development of a career identity has received renewed research interest (Haibo et al., 2017). In short, career identity refers to one’s own identity, specifically that part of one’s perception of self, that relates to work and working (Meijers, 1998, p.200), and answers the question of “what does work mean in, and for my life?” Career identity can be defined as, “a developing structure of self-concepts *in their relation to* the (future) career role perceived by the individual himself” (Meijers, 1998, p.200). This definition emphasizes the need for scholars to focus on the meaning individuals give to themselves, and the work around them. This process of giving meaning can essentially be a social process in which the individual negotiates with himself as well as with others, about how reality is to be interpreted (Bruner, 1990).

Viewed from a constructionist perspective, LaPointe (2010, p.4), defines career identity as “a practice of articulating, performing and negotiating identity positions in narrating career experiences.” Career identity is thus co-constructed in interactions. Similarly, Savickas et al. (2009), suggests a shift in career models, where the focus moves toward individual’s ongoing construction, and re-construction of subjective and multiple realities. According to the life-design counseling model of Savickas et al. (2009), self-construction and career construction, describe vocational behavior and development. In this sense, the following principles are of importance. Individuals will consider, simultaneously, all salient life roles, as they engage in career constructions. The importance of the environment is stressed, as all the roles and contexts relevant to the person, become part of the construction of their careers. For example, Ladge et al. (2015) found that more involved fathers may view their careers as less salient when they have greater childcare responsibilities, but the role of the organization (through e.g., supportive manager) plays a crucial role in fostering the coexistence of involved fathering, and a strong career identity. According to Lee et al. (2011), individuals construct careers over time, through their own sensemaking of constantly shifting, entangled strands of their personal, family, work and community lives, during which three dynamics are ongoing: external events, gradual developments, and individual actions.

## Materials and methods

### Aim

The main purpose of the present study was to explore how young working fathers experience their new family roles, and their associated responsibilities. The study investigated how this experience influences their careers, together with the influence of their work-family needs.

### Study design

The study entailed a qualitative survey (Fink, 2003), conducted from an interpretive perspective, using semi-structured interviews. Interpretive research allows for in-depth understanding of a phenomenon in context (Crotty, 1998), which allowed us to explore the meanings and value, participants attached to their family and career identities, and the intersection of these, within the context of a dual-earner relationship. The qualitative survey is designed to gather a diversity of views on a topic (Jansen, 2010), making it an appropriate approach to explore the variety of perspectives presented by the young working fathers.

### Participants

A purposive, voluntary sample of young South African fathers between the ages of 26 and 37 was drawn (Etikan, 2016). They were all in the establishment phase of their career, and purposively chosen according to the following criteria: (1) participants had to be employed, (2) be in a dual-earner relationship, and (3) have one or more children, thus being considered a working father. Participants were recruited by fieldworkers (described in more detail below) as they were of similar ages and life stages to the participants. The diversity within the group of fieldworkers allowed for greater diversity in the participants interviewed. For a more detailed description of the participants, please see Table 1.

**TABLE 1:** Overview of the sample and participant characteristics.

Item	Category	Frequency	Percentage
Language	Afrikaans	20	44.4
	English	11	24.4
	Isizulu	1	2.2
	Sepedi	4	8.9
	Sesotho	1	2.2
	Setswana	2	4.4
	Tswana	1	2.2
	Xhosa	2	4.4
	Xitsonga	1	2.2
Highest level of education	Grade 12	5	11.6
	Bachelor’s degree	20	46.6
	Honour’s degree	9	20.9
	Master’s degree	4	9.3
	Other	5	11.6
Work industry	Agriculture	1	2.3
	Automotive	4	9.3
	Chemical Engineering	2	4.7
	Construction and Engineering	6	13.9
	Communication, Media and IT	6	14.0
	Defense and military	2	4.7
	Finance	10	23.2
	Human Resource Management	2	4.7
	Manufacturing	3	6.9
	Medical	2	4.7
	Religion	3	6.9
	Transportation and Logistics	2	4.7
Work hours of fathers	31–40 h per week	15	33.2
	41–50 h per week	25	55.5
	More than 50 h per week	2	4.4
	13–30 h per week	6	14.0
Spouse’s work hours	30–40 h per week	21	48.9
	41–50 h per week	9	20.9
	More than 50 h per week	3	6.9
	Flexi hours	4	9.3
Number of children	1	22	48.8
	2	16	35.6
	3	3	6.7
	6	1	2.2
Age of children	0–3	21	52.5
	4–5	5	12.5
	6–10	10	25.0
	Older than 10	4	10.0
Making use of 3rd party assistance	Babysitter/Nanny	7	15.5
	Family member/Grandparents	6	8.8
	Domestic Helper	21	46.6
	Day-care	4	8.6

Please note that some participants did not reveal all their biographical information.

The sample included fathers who were involved in dual earner heterosexual relationships. They represented mainly Afrikaans (44.4%) and English (24.4%) speakers, even though there were participants from other language groups as well. In respect to highest educational qualification, participants were well educated and had a Bachelor’s (46.6%), Honour’s (20.9%), or Master’s degree (9.3%). In respect to the type of work, as well as the industry in which this was conducted, there was significant diversity with a broad range of sectors included. Both the participants (55.5%) and their spouse’s (20.9%) work between 41 and 51 h a week. The majority of the participants have one child (48.8%), a significant number of which are under the age of three (52.5%) and require assistance from either a domestic helper (46.6%), a babysitter (15.5%) and/or a family member (8.8%).

### Data collection

A total of 45 face-to-face semi-structured interviews were conducted by fieldworkers, and data saturation was attained (Onwuegbuzie and Leech, 2007). The fieldworkers were masters’ students, both male and female, who were trained in interviewing skills by the current authors and worked within a formalized process developed by the latter. The fieldworkers were trained in the process and given clear guidelines to which they were required to adhere. Interviews were held using a standardized interview schedule, where questions were centered around fathers’ experiences of their family roles and responsibilities, work roles and responsibilities, and the mutual influence of each, on the other. Within an interpretivist approach, the focus is on individual construction and meaning, and therefore, broader questions were constructed to elicit discussion. Example questions were: “How do you experience your family roles and responsibilities?”, “How do you experience your work roles and responsibilities?”, “Given these family roles and responsibilities, how do you think these influence your career, and the roles in your career or work?”, “Do you feel that your family roles influence your decisions, progression, and success of your career? Please explain.” In addition, to encourage deeper and more information rich responses, the use of communication techniques such as reflection, clarifying, summarizing, and paraphrasing were employed. All interviews were recorded, and transcribed verbatim.

### Data analysis

Data was analyzed by the first author, in Atlas.ti, using the steps of thematic analysis (Braun and Clarke, 2006). Firstly, interview scripts were read reflectively to gain an introductory sense of the data. Thereafter, initial coding was done, assigning codes to relevant extracts of the data. These codes were then reviewed by the second author and discrepancies discussed and resolved. At this point, relevant themes were identified, and these were tested against the extracts of the data, to confirm their viability. Finally, each theme was developed, and exemplar extracts chosen, to best demonstrate the nuances thereof. Important in this process, is the recognition of the active role of the researcher, and that the final themes rest on our interpretations and unique constructions of the dataset (Crotty, 1998; Braun and Clarke, 2006).

### Ethical considerations

Before data collection commenced, permission was gained from the ethics committee of the institution of the authors, reference number MA/2019/2. Oral and written, informed consent was obtained from each participant before the commencement of each interview. Participation in the study was voluntary and participants were made aware that they could withdraw from the study at any point in time, without negative consequences. Interviews were carried out in such a way, that participant’s comfort and privacy was always respected, for example, arranging a suitable date, time, and location for each interview. All data was kept confidential throughout the process, by means of allocating participant numbers to each interviewee. All fieldworkers involved were briefed on the research study aims, and the ethical considerations to be upheld.

### Trustworthiness

To obtain trustworthiness of the data, we drew on the guidelines presented by Miles and Huberman (1994), to ensure the criteria described by Guba and Lincoln were met (dependability and transferability). To ensure dependability, and the potential threat posed by multiple field workers, detailed training was provided, and interviews were conducted according to a strict protocol and question schedule. To maximize transferability, a diverse sample was drawn on, which included participants from a variety of ethnic backgrounds, ages (within the target group), industries and job categories. These are described in more detail in Table 1. Finally, credibility was maintained through the involvement of both authors in the analysis of the data.

## Results

Emerging from this study were three main themes, each with several subthemes, which will be discussed below, with the aid of extracts from the participants’ interviews. The three main themes were: “the meaning of family identity,” “the impact of family identity on career identity,” and finally, “the types of negotiation scenarios” used by these dual-earner couples, in balancing the work-family challenges they face. Table 2 illustrates the themes and sub-themes as constructed from the data.

**TABLE 2:** Themes and subthemes of young working fathers’ family and career identities.

Themes	Subthemes
The meaning of family identity	Shift in identity hierarchy Roles of young fathers
Impact of family identity on career identity	Deliberate life design Career related decision making Temporal considerations in balancing career and family identity
Types of negotiation scenarios	Traditional but flexible Tag team Non-conventional

### Theme 1: The meaning of family identity

#### Shift in identity hierarchy

Most participants in the sample had children below the age of 10 years, with a large proportion of these, 5 years and younger. In most instances, new father’s displayed considerable excitement over the birth of their child and highlighted the changes this had wrought in their lives. These participants suggested that the importance of family identity appeared to be triggered by the arrival of the couple’s first child, and the anticipation of fatherhood and its responsibilities. Whilst their significant others were important to them, the expectation of fatherhood led to a shift in their identity hierarchy. While the couple was arguably a family before this time, they were both responsible adults, both working and could care for themselves. Thus, it was the arrival of the first child, and the responsibility this entailed, that caused a shift in the father’s *priorities* and *motivation for working*.

*“At first, when you think that we’re pregnant you think that nothing is going to change, but your whole career-driven side kind of takes a backseat. Your family comes first. So, whatever you do in your career now, it is so that you can look after your family better. It’s not to enrich yourself anymore when you say, ‘I’m going to achieve this in my life.’ I’ve essentially got to a point where my main focus has shifted and that only happened after she was born. I essentially just want to be a good breadwinner. Other than that, it shifts to the family because that is my main role now. It’s not whether I’ve got ambition, or I want to be an MD. It doesn’t go away, but it’s not as high on the priority list as it was” (P10).*

Interesting to note in this extract, is the reference to “we” in respect of the pregnancy, terminology used by many of the young fathers. In contrast to pregnancy being something limited to their wives, it is considered a joint effort and responsibility, with both the mother and father “giving birth” so to speak. Notable here too, is the shift in priorities in respect of the importance of career. While career goals remain in place, their importance has shifted, and they are no longer the priority. Several participants made mention of this shift which happened, not on the commencement of their identity as a couple but at the onset of fatherhood, and the genesis of this identity. The underlying purpose for career goals has shifted and these are no longer in service of themselves, but rather to take care of the family as a whole. While their career identity remains central, its position in the hierarchy of importance has shifted.

The arrival of parenting meant that the participants’ lives and activities were effectively rearranged but despite the challenges associated with parenting, they believed it was a source of great joy and made one a better person, as it encouraged selflessness and built character. Moreover, it was associated with greater maturity, something which they also believed inadvertently impacted their career identity in a positive way. Fatherhood, although associated with great joy and pleasure, required wisdom and responsibility. Because children are only in one’s care for a while, they believed that this time should be utilized wisely, and this explains the shift in the identity hierarchy.

While fathers of older children did not necessarily share the excitement attached to the arrival of a new child, especially the firstborn, their commitment to their fathering identity, through active involvement in their children’s lives, was consistent throughout the sample. This manifested through their concern for providing for their children, ensuring a strong moral example, building relationship with their children, helping with homework, attending sports matches, taking children to extra-murals, and spending leisure time with their children, engaged in suitable activities. While the degree of centrality was influenced by the type of negotiation scenarios decided on by the couple, their father identity was something they took seriously and placed considerable value on.

Moreover, participants, who came from diverse backgrounds, suggested there were generational differences in parenting styles because of societal changes in cultural and traditional patterns. Many of them reflected on the differences in their family structures and roles in comparison to those of their parent’s generation. These comments highlighted the significant shift in attitudes around family, and the centrality of the father’s involvement.

*“I wouldn’t say that it come from someone specific, but I think it’s part of the older generation’s way of doing. I suppose it also stems from how society worked 100 years ago. There will be a husband that worked in the field or on the farmland or have a job and then there was a housewife. I suppose it was the normal workings of everyday life. Things have changed dramatically, so where you have a husband and wife working both 8 to 5 jobs, and it’s just not a sensible way of doing things anymore” (P43).*

Identity is inherently social and is drawn *inter alia* from group and interpersonal relations (Alvesson et al., 2008). In constructing their father identities, participants renegotiated their fathers’ patterns, and strove to both to emulate and distinguish themselves from these. Some had experienced their own fathers as distant and uninvolved and thus, strove to construct their own father identity in a very different way. Others drew on experiences of their own fathers in making sense of their father identity. Participant 38 mentioned that his own father had been very absent and uninvolved, due to having no father figure himself. This lack of relationship had clearly affected him negatively and he was very deliberately cultivating a very different fathering identity.

*“There was no relationship. So, he did the best he could. And what he knew. I decided, listen I want to be there. I want to be involved in every single area of their life.* … *And it’s just creating that open relationship with your children, being involved, knowing and being involved in every small detail of their lives. I don’t know, it’s just something I*…. *that’s the type of father I want to be and I want to be involved. I want to be there, I want to know, he can approach me anytime with any sort of issue” (P38).*

#### Roles of young fathers

Having considered the shift in identity hierarchy for participants, we will now consider the roles these fathers perceived to be central to their family identity.

Table 3 provides an overview of the main roles of working fathers identified in the data, and the activities associated with these. In keeping with the nature of the qualitative survey, which is to explore diversity, all the roles mentioned by participants have been included. The numbers in brackets listed after each role provide an indication of the number of participants who alluded to a particular role at least once, as described by the descriptors in the table. The aim here, is not to repeat the list but rather highlight a few aspects of interest. Firstly, even though participants were in dual-earner couples, nearly all the participants felt that a primary role was to be the breadwinner of the family, irrespective of the fact that their wives were working and, in some instances, earning more than they were. This role, more traditionally associated with fathers, aligns with roles and characteristics identified by Humberd et al. (2014), McLaughlin and Muldoon (2014), Ladge et al. (2015), who highlighted the importance of the provider role. In our data, it was the perceived need to provide for their families, that provided impetus for the shift in the identity hierarchy mentioned above, the reason for which will be explored in the next section.

**TABLE 3:** Roles of young working fathers.

Role		Activities associated with the role
Provider (32)		Father as breadwinner, income manager, administrator, provider of education
Leader (21)		Father as coach, disciplinarian, setting boundaries, providing guidance, present and engaged, responsible for family well-being and support
Protector (10)		Father as responsible for security and protection, keeping the family safe, likely context bound due to the context of SA
Role model (8)		Father being an example and conscious of what he says and does
Domestic role	Household chores (24)	Related to the smooth running of the house, includes activities such as gardening, cooking, grocery shopping, taking care of cars and the yard
	Child-care chores (29)	Bathing children, changing nappies, taking children to school, managing extra-murals, staying at home when children are sick, helping with homework

*“I do want to spend time with my family and my kids*…*but the thing is you need to provide, you need to make sure there’s bread on the table. There’s food on the table, a roof over their head. There’s water. There’s everything. So, you need to make sure there, your finances are*… *fortunately my wife and myself we are in a blessed situation where we’ve both got work and we don’t earn bad salaries” (P8).*

Secondly, many of the participants viewed the father identity as associated with leadership in the family, though this was not interpreted as a distant authoritarian figure but presented as a figure akin to a servant leader. The leadership role was presented as someone who provides guidance and direction, is present and engaged, and cares for the wellbeing of his family. The characteristics identified in this role align with aspects of a good father identified by Ladge et al. (2015) namely, providing discipline, being involved and being a leader, guiding and coaching (p.160). The leader role was perceived to bear great responsibility, and fathers were aware of the impact their decisions and conduct had on the family unit.

*“The thing that pops into my head is to be a good husband, obviously I need to spend time with my wife, a good husband and then a good father to my children. I think it’s important to lead my family in the right direction, because where I go, they go as well. And whatever decision I make, has an impact on their lives. So, I need to make a decision that’s good for everyone. Otherwise, it will have a repercussion on everyone” (P45).*

The role of father as protector was an interesting one, and may be largely due to the instability of the South African environment, the high crime rates and the fear fathers have for the wellbeing of their families. It does however, highlight the role of socio-cultural factors in constructing and making sense of identity (Alvesson et al., 2008; Stryker and Burke, 2022). The father as a/the role model, also identified by Ladge et al. (2015), involved providing an example in all areas of life, both work and family, as suggested by participant 6. This also highlights the interdependence of the family and career identity.

*“A big part of family success is being that example of a successful businessman as well. I want to portray that kind of role model for my kids. To be a business leader I want them to be ambitious. I want them to develop their own skills and push and see whatever they can achieve in life that kind of things. So, family success is also dependent on work success. It goes both ways I want to be the role model to my kids as a father who was able to do business and then do family at the same time” (P6).*

The final role mentioned by the participants related to the domestic sphere and responsibilities in this domain. These were divided into household chores and child-care, and all the young fathers took responsibility for chores in both domains, though the degree to which this was done was dependent on the arrangements between the spouses, something we will discuss in theme 3. A similar theme is mentioned by Ladge et al. (2015) as an aspect of being a good father, though this is limited to child-care tasks, whereas the participants in our study highlighted both child-care and general domestic chores, as part of their responsibilities.

### Theme 2: Impact of family identity on career identity

In the sections below, we discuss the key areas in which family identity was perceived to impact work and constrain career identity. We have termed these deliberate life design, career related decision making and temporal considerations in balancing career and family identity, each of these will be discussed in more detail below.

#### Deliberate life design

Evident in this regard, was the intentionality several participants and their spouses had displayed, planning their work circumstances ahead of time to accommodate their family responsibilities and identity, suggesting a deliberate life-design process (Savickas et al., 2009). One participant, had created a business, based at their home, accommodating both his and his wife’s career interests, to allow them to integrate their work and home lives more easily. The business ethos was based around a family ethos with a fair amount of integration between the spaces. Another participant explained that he and his wife had both taken a step back in their careers to allow for a more hands-on approach to parenting. They had structured their working time so that both parents were responsible for raising their daughter.

“… *that was an intentional decision because we wanted to be hands-on. Hands-on also means that it is not so easy for other to come in and support you. Uhm, and so a lot of the responsibility has between the two of us, so- while I work, she looked after her and when I get home she goes to work. And so, so very hands-on and we didn’t-I didn’t want to be of those dads where work all day, uhm, with the rationalization that I bring the home the money and that was never sufficient, uhm, and so we’ve got a partnership with raising our daughter and, uhm you know, I take a lot of family responsibility leave, uhm, which I feel guilty about because it is not normally the farther who takes it.”*

He explained that he and his wife took turns working throughout the day, leaving the other parent to care for their daughter. He left for work quite early in the morning and was home around four, after which she started, from late afternoon through to the evening. This was made possible by the nature of her work – she is a psychologist and there is a demand for her services after hours. This arrangement did however, come at a cost for his career, as he was not always around for late afternoon meetings nor able to display the kind of visibility that led to maximum career mobility. Despite the impact on his career prospects, he was satisfied with his decision as he and his wife were driven by a strong value orientation (Meijers, 1998; Greenhaus and Powell, 2012) in respect of the parenting style they wanted for their daughter.

#### Career-related decision-making

Career related decision-making was influenced by the drive to ensure flexibility, support, family financial security, and reduce excessive risk-taking. Each of these will be elaborated on in the section that follows.

Career opportunities were evaluated to strategically accommodate (Ranson, 2001) the degree of flexibility they would give, with one participant suggesting that he would even be willing to sacrifice aspects such as job satisfaction, to ensure that his family life was satisfactory.

“… *so it’s one of the main things that I look at when I have considered taking other options, taking other work opportunities and stuff. The work environment that I’m in currently and the flexibility I have, is almost paramount to me*…… *But as I said, my decisions are based on my family and family time, and stuff like that. So, I’m happy to almost be a bit dissatisfied with my work environment, if I know that when I get home I am satisfied with my family” (P4).*

Participant 32 for example, highlighted his deliberations in respect of his next job move which would require significant investment in the amount of time spent at work, something which he was not willing to accommodate as it would spill over into family time.

*“And I know, so to make it practical, I would never take a management job now because I don’t have the resource and hours to afford that to the team. Uhm, so you know, you talk about career progression I think you start eliminating opportunities you would have considered in the past because you know they would not fit into your life so if I look at the one layer above me and the role of the job and the amount of time it demands and the response it demands and how many meetings run late into the evenings, it is just not possible” (P32).*

Several participants mentioned receiving lucrative job offers from other companies, but these would require considerable travel, both nationally and internationally, and whilst the offers represented excellent career opportunities, they were not prepared to sacrifice family time, or place the full responsibility for domestic and child-care onto their wives. In the extract below, Participant 18 highlights the importance of a father’s financial, emotional, and practical support within the family, both in respect of his children and his wife.

*“I take care of 50% percent of the home responsibilities, if I leave who will take care of that? No, as much as my career is important so is my family*… *you can’t leave one aspect of your life*…*they have to move in parallel. I can’t leave my wife to take care of the home and children alone*…*Would I just be the father that provides on my money? No, my idea of being a father includes being present and participating and being involved in my kid’s school and sports activities. I want to be involved in my kid’s life*…. *If I focus on career progression, my family might suffer*… *No, I prefer to take jobs that will still provide me with flexibility and the opportunity to be there for my family*… *I also provide emotional support, I have to be there*… *I want to be there when my wife comes home after a long day (P18).*

Participants suggested that family financial stability took priority, and decisions were also made to ensure their families would be secure. Even though many of their wives were also earning good salaries, the traditional role of fathers as breadwinners, still played a central role in the construal of their family identity, and thus impacted their career-related decisions, in respect of the centrality of this perceived role.

*“Yes, definitely, if I can just give you an example, when I finished my degree, I wanted to go through a stream of becoming a conveyancer, but it is a long route. So I decided, that you know what with the burden that I have of the family that I needed to take care of, let me try to get work so that I can take care of my family, so, somewhere, somehow, I can say, my decision to look for work instead of following the career path that I wanted, it was exacerbated by the need of taking care of my family. So, obviously, they influence my career path” (P6).*

Related to the financial aspects, were career decisions involving risk, which was particularly relevant in the entrepreneurial ventures in which some of the participants were involved. In contrast to their days of being single and even a couple, opportunities that were considered too risky were turned down as they did not want to jeopardize their family’s security.

*“I think the decisions that you take then are high level and risk-taking decisions, where it’s about uh, extending your resources to take on more work, you consider your family role and your family situation in terms of, is it too risky financially, or time-dependent, you would think a lot harder and a lot further, before pulling that trigger” (P9).*

#### Temporal considerations in balancing career and family identity

In respect of balancing career and family identity, participants alluded to two timeframes, those which involved long term consequences and those which involved considerations over the short term. In respect of long-term decisions, there was a focus on all the parties involved and how their needs could be accommodated, with partners taking turns over time to sacrifice or indulge in their interests, for example, taking turns to engage in additional studies. Long term decisions involved a careful weighing up of pros and cons for all involved, taking into account the impact on the family as a unit as contextual factors of relevance.

“…*the first thing we looked at is, one: what is happening in the country as a whole. What are the expectations out of women being young, being black, being educated, being able to perform? Who will stand the best chance in terms of getting growth and who enjoys the sales environment more? And I took the obvious choice of personally I’m not really cut out to be a salesperson*… *I can do it and I have been successful at it but that’s not my preference. That she stays in sales, it’s something she enjoys and she is good at, it’s something I could do and I was good at but hated doing*… *So how do we push her in the right direction and her ambition and I play more of a supporting role in terms of finances and that” (P17).*

*“Yeah, I would love that. I would love to finish everything that I start, but uhm I just hope that at that particular time I would still have the energy or I would still have the necessity. I’ll be like forty five/forty six perhaps I’ll have a few years left to complete my second degree. But uhm its always an option that I will explore when we get there, but where we are at the moment, I don’t have that kind of time to do the studies” (P28).*

In addition to having to make long-term decisions, there were also short-term decisions to be made to manage their various responsibilities on a day-to-day basis. Central here, was the importance of flexibility, something already highlighted earlier in our discussion. Other elements which facilitated this process were teamwork, both at home and at work, an accommodating supervisor, management by objectives rather than a focus on specific working hours, the importance of planning ahead and determining priorities, effective time management and the level of the job. Most South African companies do not have formal paternity leave arrangements, and this was mentioned as something which would have been of support, especially in their early efforts in managing work-family tensions.

In respect of the short-term time frames in balancing work-family demands, we identified three broad types of negotiation scenarios, discussed as the next theme.

### Theme 3: Types of negotiation scenarios

Frear et al. (2019) highlight the need for understanding the social processes occurring at home as dual-earner partners negotiate the work-family interface. We identified three broad types of negotiation scenarios, which we have termed traditional but flexible, tag team and non-conventional. These scenarios describe the ways in which young, dual-earner couples manage the responsibilities and demands of both work and family. In managing these challenges, several participants indicated that they were assisted by support from family members, domestic workers/nannies, as well as nursery schools or creches. In line with the nature of the qualitative survey, which is to explore diversity, the negotiation scenarios presented here represent broad types, with considerable diversity within each. These are presented as a continuum ranging from most to least conventional. However, the majority of participants can be described as engaging in the tag-team style though there were several examples of traditional but flexible and non-conventional types as well.

#### Traditional but flexible

The traditional but flexible scenario was closest to the traditional domestic roles with the father as the primary breadwinner, and mom as the primary caregiver, though often with the support of a helper or nanny. This was usually because the mother’s work demands allowed for more flexibility to accommodate her desire to be more involved in her child(ren)s’ life(ves). Other factors which influenced this was the nature of work (for example being a business owner), geographical location of work in relation to home, and the flexibility to work from home or not.

*“No, in a general set up she’s more flexible with her time being an estate agent, but that also means that she has to take clients who work during the day and want to see a house at night. So around 6 my son wants to eat and if I’m there, I’ll make the food, and when she’s there she’ll make the food or give the food or whatever the case is” (P42).*

*“But already when my daughter was born, my wife went to her company to say: Listen, I can only work half day which they accepted. So, we have already thought of that many years before she went to primary school. So that my wife can pick her up after school, assist her with homework she might have and then hopefully I will be in a position where I will be more at home and as I said to be able to dictate my time. So, I will be able to say: Listen from 7 till 2 it’s all work and then from then in the afternoons I will go home to assist my daughter with everything” (P 37).*

Despite these father’s leaving a greater share of the household and child-related chores to their wives/partners, they nevertheless were actively involved in their children’s lives, often finding a specific activity each day they would engage in, for example, being responsible for bath time or opting to attend sports games.

*“First of all, my wife understands the finance industry, there are deadlines. Like now, we have hearings, so now every night we’re pushing the hours. She understands it, but when my son was born, I made a decision to be involved, because there will*… *you know, there will always be another day to sort out a problem at work. But there will be only one first rugby game, one first cricket game, one first whatever, so, my first day of school. I will be involved in all of that. So, what I have done from when he was born was, like bath time was me and his time. So, I made sure that I’m at home at between 5 and half past 5, because that’s the time for him to bath. So, I did that. My wife didn’t do that. Just to connect and for us to have a relationship” (P 42).*

#### Tag team

In the tag-team scenario, both partners worked relatively demanding jobs and were responsible for domestic and childcare chores, with an emphasis on co-parenting. Fathers were actively involved in doing the cooking, grocery shopping and a variety of child-related activities such as extra-murals, homework, and taking children to school. The allocation of roles was negotiated between partners in various ways, and differed between the couples surveyed. In some instances, roles were not clearly defined before-hand and were negotiated on a moment- by- moment basis, based on the needs and practicalities of the situation and the availability of partners to meet those needs.

*“I do not think there is a defined role. I do not think there is something specific – the dad does this, or the dad does that. I think in our household we are absolutely a tag team and what is uhm*… *necessary at this stage is who can take the easiest lead or responsibility. There are no designated roles, I think we more adapt to the specific needs of the children and the specific needs of how we are wired, anatomically and biological clocks and things like that (P 34).*

In other instances, the allocation was driven by partners preferences, strengths and/or, the desire to exercise or indulge hobbies.

*“I mean we share everything*… *I mean I shared nappies, changing clothes, uh packing her bag for school, to run household chores, I mean doing the dishes, I do most of the cooking in the house [laughs]*… *So, I’ll cook everyday of my life, so I don’t really mind at all. Um so ya I do most of the cooking, my wife does most of the cleaning again. Yeah, but she- it’s not that she needs to clean, it’s that she just wants to clean. She loves cleaning.” (P20).*

In addition, *ad hoc* responsibilities were negotiated on a week to week or day-to-day basis, depending on each parties needs and immediate work pressures. The tag team approach meant that both parents took family responsibility leave, took turns to work at home and at times, worked late at night or very early in the morning to finalize urgent work. This did however, require constant planning to ensure that the multiple demands were met as well as constant open communication between partners. Moreover, it involved sacrifice on behalf of the fathers, an attitude of maturity, discussed earlier, and in some instances, being prepared to depart from traditional cultural patterns.

#### Non-conventional

In some of the couples, the traditional roles were slightly reversed and while both parties were working and breadwinners, the father took on a greater domestic and child-care load than his wife, often in conjunction with a helper. Like the more traditional scenario, this arrangement was based on the nature of each partners’ work and the degree of flexibility it offered, work time schedules, work demands and responsibilities, geographical location, and travel time to and from work.

*“We wake at about 6:30 the kids wake and then with my wife working at Sandton and me working very close to home. It’s my responsibly at this stage to get the kids ready for school. Get the food for the day. So we make breakfast, we make the food for the day, get the kids in the car, get the kids to the school. Then go to work myself. Then in the afternoon I go and fetch them. Bring them back home and then make dinner” (P7).*

Some of the wives in the sample had particularly demanding and stressful jobs, including managerial responsibilities, which meant they were simply unable to share a 50–50 domestic load. For some participants this was considerable change from their natal families, while others followed a similar pattern to their own parents. In this scenario, it was the father who managed most of the extra-murals, schoolwork, and household chores.

*“She’s got a very stressful job, so*… *she’s a very senior person in her company. I think the difficult thing for our household is, because she’s got such a demanding and stressful job, and she’s constantly busy with work. Even when she’s home, her bosses will phone her and she needs to run to her laptop quickly and do things, so then I have to look after the kids. So ja I probably have more kid’s responsibilities than most dads do” (P44).*

This put considerable pressure on the fathers involved and led to conflict at times, as some of them struggled to balance work-family demands. Moreover, the additional family pressure and the lack of freedom this implied, compromised their ability to indulge in “man-things” (P 44). This was however justified by the time they could spend with the children, and the impact this would ultimately have on them.

## Discussion

The framework of FRWD, suggests that individual’s decisions in the work domain are influenced by their family situation (Greenhaus and Powell, 2012). This study provided strong empirical support for this view, highlighting the roles of working fathers, as indicative of their family identities and how these then influence their career decisions. Furthermore, our findings shed light on ways in which dual-earner couples negotiate their work-family needs in order to foster positive work-family outcomes (Greenhaus and Powell, 2012). Our findings resonate strongly with the two important notions of the FRWD, namely (1) that the decisions men make at work are influenced by their family situations/identities and (2) these decisions are made in conjunction with their partners (Challiol and Mignonac, 2005; Greenhaus and Powell, 2012).

In respect to the first notion of FRWD, our themes 1 and 2 have relevance.

Our first theme describes the different family-related roles young fathers adopt, namely breadwinner, leader, provider, protector, role model and household and childcare roles. Although some of these roles do correspond with previous literature (Humberd et al., 2014; Ladge et al., 2015), working fathers in our sample, described their role as the protector in relation to their family, as well as their responsibility for household-chores, as unique. In this sense, our description of the family roles of working fathers provides a more nuanced explanation of how they, not only profess the need to be more involved, but have a strong desire to play a more active part in their family’s lives. Participants suggest a strong value-orientation and strategic life design, in relation to their family-related career sensemaking (Meijers, 1998; Savickas et al., 2009). Furthermore, our findings support the idea that, the more strongly fathers identify with their family relationships, the more concerned they will be that work decisions benefit their families (Greenhaus and Powell, 2012). As such, family identity was shown to influence work related decisions, especially in respect to the direction and progression their careers could take. Of relevance, were career-related financial decisions and their impact on the family, the degree of risk they were prepared to expose their families to, and the type of work and opportunities they would consider. In this regard, our findings provide support for a shift in the identity hierarchy, with the family identity enjoying increasing saliency, as suggested by Rothbard and Ramarajan (2009). Thus, when making career-related decisions, our sample, in almost all instances, suggested putting their family’s needs first. Lee et al. (2011), show how managerial employees’ careers unfold over time, in relation to family and personal life developments. Our findings suggest this is indeed the case, but it is the shift in the saliency of identities that triggers the influence on their career decisions. A specific example of this increased family saliency was the birth of their first child. An additional consideration was their awareness of their wives’ work demands and careers, and their need to support and encourage these. This resonates with the idea of spousal career support (Lysova et al., 2015), and spousal career aspirations (Pluut et al., 2018). Both concepts are considered extensions to the FRWD and were supported by our data.

Lee et al. (2011) draw attention to the importance of a temporal element, in the interactive development of peoples’ lives and careers. When considering support in the context of dual-earner couples, our findings revealed that career decision making takes place over two subjective time frames (Mayhofer, 2020), namely over the long term and the short term, both of which are negotiated between partners. In this regard, our findings contribute to temporal career theorizing in respect of the FRWD, by incorporating a temporal dimension (Mayhofer, 2020), when explaining the negotiations of work-family needs between dual-earner partners.

When considering the temporal dimensions, and more specifically, the negotiation of work-family needs over the short term, in theme three, we identified three broad types of negotiation structures. These are traditional but flexible, tag team and non-conventional. These structures were influenced by both partners’ work circumstances, demands, geographical location of work in relation to home, travel time and working hours. As suggested by Harrington et al. (2016), traditional gender expectations continue to exert an influence on family structures, and this was demonstrated in the traditional, but flexible negotiation structure. This negotiation type reflected traditional roles, though nevertheless, entailed an actively involved father, especially in relation to child-related chores and responsibilities.

With regards to the tag team negotiation structure, both partners were actively involved in co-parenting (McLaughlin and Muldoon, 2014), and attending to household demands and chores. These were negotiated in respect to partners’ preferences, strengths, practicalities of the situation, work responsibilities and hobbies. This type of negotiation structure is very similar to the egalitarian fathers identified in the millennial research of Harrington et al. (2016).

Finally, we identified the non-conventional negotiation structure, which involved co-parenting but in which fathers had an increased domestic role, due to the demands and circumstances surrounding their wives’ employment. In contrast to a stay-at-home dad (Schwiter and Baumgarten, 2017), the fathers in our sample worked fulltime (more than 35 h a week) in addition to their household and childcare responsibilities. Both these types of negotiation structures were made possible by the shift in identity salience for working fathers discussed earlier. This is in line with the recent findings of Erdogan et al. (2019), that suggests that “organizing work and family roles in a salience hierarchy with a predominant non-traditional gender role is associated with less work-family conflict, especially for women” (p.1778).

Overall, our findings in respect of young working fathers support Lee et al. (2011), in their assertion that people are not powerful autonomous agents, but rather relational beings whose careers are shaped in a network of interdependent relationships (p.1549).

### Limitations and future research

Our study was purposefully focused on a subset of men (working fathers in dual-earner relationships), specifically those within the establishment phase of their careers, as young working fathers remains a topic which warrants further research (Ranson, 2001; Ladge et al., 2015). However, investigating working fathers in other career stages of Super’s career stage model (e.g., the maintenance), might provide for different identity dynamics. For instance, fathers in their maintenance phase, might reveal how their parenting responsibilities and demands decrease, as children become more self-sufficient and require less care. In this regard, it might be that fathers in this phase have more time to invest in their careers, and their career-related decisions are less likely to be influenced by their family identities.

It is possible that the education level and the nature of the work for some of the participants (e.g., business owners) has skewed the degree of involvement in the participants’ children’s lives. We suggest that this occurs due to the increased flexibility that our sample have in their working environment as time lost during the day can be made up after hours. So in this regard, it might be the combination of education and flexibility that allowed for greater participation in addition to participants deliberately choosing work that would allow them the requisite flexibility. This has been described in the literature as a characteristic of Gen Y employees.

An aspect worth mentioning, which might have impacted the current findings, is that data was collected in 2018/2019, prior to the COVID-19 pandemic. This is relevant in light of recent studies, which have explored the impact of COVID-19 on work-family needs and circumstances and illustrated the changes in views regarding the world of work and its relation to employees’ personal lives (Guan et al., 2020; Lee, 2021).

Additional areas of future research could explore our findings in terms of the three negotiation scenarios further, by validating this tentative typology, in a more quantitative manner. This can provide for more valid comparisons of fathers with different negotiation styles and the impact thereof, on their careers.

## Conclusion

As suggested by Cabrera et al. (2018), a persistent challenge in research is its consistent focus on mothers, as children’s primary caregivers. Our study attempted to rectify this imbalance, by considering the role of working fathers in relation to their work-family needs, and how these shape their career identities, work experiences, career progression, or adult career development. In this regard, our overall findings provide evidence in support of the framework of the FRWD. More specifically, how the family identity of young working fathers influences their decision with respect to their careers, while negotiating the work-family interface with their dual-earner spouses.

### Implications for practice

Our findings reaffirm the views of Humberd et al. (2014), that although there has been a shift in the saliency of family identity amongst young working fathers, such views are not necessarily reflected in the organizational structures or culture. In this regard, the importance of a supportive work-family culture in organizations should be emphasized, in support of both men and women as they pursue their careers. This implies the need for gender neutral work-life policies and programmes, as well as interventions in organizational culture which could include a shift in leadership attitudes toward working fathers (Humberd et al., 2014). From a more practical perspective, the information generated in this study can be used as a guide or starting point of discussion by career counselors, in their consultations with working fathers, who experience conflicting demands during the establishment phase of their careers.

## Data availability statement

The raw data supporting the conclusions of this article will be made available by the authors, without undue reservation.

## Ethics statement

The studies involving human participants were reviewed and approved by Ethics Committee of the Department of Human Resource Management, at the University of Pretoria (reference number: MA/2019/2). The patients/participants provided their written informed consent to participate in this study.

## Author contributions

Both authors participated in the design and coordination of the study, as well as the editing of the manuscript, supervised the collection of data (overseeing the fieldworkers) as well as the analyses thereof, had equal contribution to the drafting of the manuscript, read, and approved the final version.
